# The enzymatic de-epithelialization technique determines denuded amniotic membrane integrity and viability of harvested epithelial cells

**DOI:** 10.1371/journal.pone.0194820

**Published:** 2018-03-27

**Authors:** Peter Trosan, Ingrida Smeringaiova, Kristyna Brejchova, Jan Bednar, Oldrich Benada, Olga Kofronova, Katerina Jirsova

**Affiliations:** 1 Laboratory of the Biology and Pathology of the Eye, Department of Paediatrics and Adolescent Medicine, First Faculty of Medicine, Charles University and General University Hospital, Prague, Czech Republic; 2 Laboratory of the Biology and Pathology of the Eye, Institute of Biology and Medical Genetics, First Faculty of Medicine, Charles University and General University Hospital, Prague, Czech Republic; 3 Institute of Microbiology of the Czech Academy of Sciences, Prague, Czech Republic; Cedars-Sinai Medical Center, UNITED STATES

## Abstract

The human amniotic membrane (HAM) is widely used for its wound healing effect in clinical practice, as a feeder for the cell cultivation, or a source of cells to be used in cell therapy. The aim of this study was to find effective and safe enzymatic HAM de-epithelialization method leading to harvesting of both denuded undamaged HAM and viable human amniotic epithelial cells (hAECs). The efficiency of de-epithelialization using TrypLE Express, trypsin/ ethylenediaminetetraacetic (EDTA), and thermolysin was monitored by hematoxylin and eosin staining and by the measurement of DNA concentration. The cell viability was determined by trypan blue staining. Scanning electron microscopy and immunodetection of collagen type IV and laminin α5 chain were used to check the basement membrane integrity. De-epithelialized hAECs were cultured and their stemness properties and proliferation potential was assessed after each passage. The HAM was successfully de-epithelialized using all three types of reagents, but morphological changes in basement membrane and stroma were observed after the thermolysin application. About 60% of cells remained viable using trypsin/EDTA, approximately 6% using TrypLE Express, and all cells were lethally damaged after thermolysin application. The hAECs isolated using trypsin/EDTA were successfully cultured up to the 5^th^ passage with increasing proliferation potential and decreased stem cell markers expression (*NANOG*, *SOX2*) in prolonged cell culture. Trypsin/EDTA technique was the most efficient for obtaining both undamaged denuded HAM and viable hAECs for consequent culture.

## Introduction

The human amniotic membrane (HAM) is the inner layer of the fetal membranes. It consists of a single layer of epithelial cells, basement membrane (BM), and an avascular stroma [1]. The two cell types of different embryological origin are located in the HAM: human amniotic epithelial cells (hAECs) derived from the embryonic ectoderm, and mesenchymal stromal cells (hAMSCs) derived from the embryonic mesoderm [1].

The wound healing effect of HAM mediated by numerous growth factors and cytokines and the presence of stem cells continuously increase interest in its potential in the medical treatment and tissue engineering [2–7]. The application of HAM is best established in ophthalmology, where it is used clinically for its wound-healing effect and as a substrate for limbal stem cells (LSCs) cultivation and consequent treatment in limbal stem cells deficiency (LSCD) [8].

Many published reports discussed whether intact or denuded HAM is more suitable for culture of LSCs. It has been shown that intact HAM mostly supports the growth of limbal explants [9–11], while denuded HAM is more suitable as a substrate for enzymatically dispersed LSCs [12–17]. Koizumi et al. found that denuded HAM supported the growth of well-stratified and differentiated LSCs, while on intact HAM a monolayer of less differentiated limbal cells was formed [18]. LSCs cultured on denuded HAM were better attached to the stroma [18].

The expression of stemness genes, e.g. octamer-4 (OCT-4), sex determining region Y-box 2 (SOX2), fibroblast growth factor 4, zinc finger protein 42 (REX-1), nanog homeobox (NANOG), ATP-binding cassette sub-family G member 2 (ABCG2) and bone marrow stromal cell antigen-1 (BST-1), was reported in hAECs [19]. The hAECs have highly multipotent differentiation ability and could be differentiated into all three germ layers [20]. Furthermore, these cells have immuneprivileged characteristics, expressing only very low levels of class IA and II human leukocyte antigens [21]. The ability to differentiate, low immunogenicity and anti-inflammatory effect indicate their potential to be used in the treatment of a various diseases and disorders, such as the treatment of Type I diabetes [22] or cardiovascular regeneration [23]. The hAECs can also be utilized for tissue engineering of skin [24] or as a feeder for expanding of various stem cells types, including human LSCs [22], or human and murine embryonic stem cells [25, 26]. Li et al. found that supernatant from hAECs inhibited the chemotactic activity of neutrophils and macrophages as well as reduced the proliferation of T and B cells after mitogenic stimulation [27].

Denuded HAM and hAECs can therefore be used separately for various purposes. Several approaches and methods exist to denude HAM. The most frequently used method is treatment with the trypsin/ethylenediaminetetraacetic acid (EDTA) [28, 29]. Besides that, sodium dodecyl sulphate (SDS) [30], Tris/EDTA followed by incubation with SDS [31], Tris/EDTA/aprotinin [32], EDTA [18], thermolysin [33], dispase [14] NaOH [34], or ammonium hydroxide [35], were successfully used.

The best established method for the isolation of viable hAECs is the trypsin/EDTA treatment [36–40], and its modified forms like several trypsin/EDTA incubation steps [41] or treatment with dispase [42, 43].

Each of the mentioned techniques has different effects on biological and physical properties of both HAM and hAECs. Many of these treatments take hours and may damage denuded HAM integrity, or viability of hAECs and hAMSCs or decrease the activity of growth factors. EDTA itself does not remove epithelium completely [14, 17], treatment with dispase can damage BM structure [13]. However, these studies were focused on either de-epithelialization or on obtaining of viable hAECs only.

In this study, TrypLE Express, trypsin/EDTA and thermolysin were applied to obtain both viable hAECs and undamaged denuded HAM at the same time. TrypLE Express is a recombinant fungal trypsin-like protease with similar dissociation kinetics to porcine trypsin, which has been successfully used for dissociation of human pluripotent stem cells [44]. Trypsin/EDTA application is generally used to detach seeding cells from the culture flask and for de-epithelialization of HAM [13, 36, 39]. Thermolysin is a zinc neutral, heat-stable metalloproteinase isolated from the *Bacillus strearothermophilus*, and it has been demonstrated that its use generated fully denuded HAM without any mechanical scrapping [33].

The aim of our study was to identify an enzymatic method which would result in two simultaneous advantages: 1) a complete HAM de-epithelialization safe for BM and stroma, and 2) harvesting viable hAECs usable for subsequent culture.

## Materials and methods

### Tissue

The study followed the standards of the Ethics Committee of Motol University Hospital, Prague and the General Teaching Hospital and 1^st^ Medical Faculty of Charles University in Prague, and adhered to the tenets set out in the Declaration of Helsinki. Twelve term human placentas were obtained after the delivery by elective caesarean section (with donor informed consent) from the Motol University Hospital, Prague (study EK-503/16 approved on 04/14/2016). The donors were tested negative for hepatitis B, C, syphilis, HIV, and with CRP less than 10 mg/l. Each placenta was immediately placed in a sterile container filled with Hank´s Balanced Salt Solution without calcium and magnesium (HBSS, Sigma-Aldrich, St. Louis, MO, USA). Special attention was paid to the gentle handling of each placenta during procurement, transport and subsequent manipulation. The preparation of HAM started at most within 2 h after the delivery. HAM was mechanically peeled off of the chorion and washed several times with HBSS to remove blood clots and debris. HAM was flattened onto a sterile nitrocellulose membrane (Bio-Rad, Hercules, CA, USA) with the epithelium surface facing up, cut into 2 x 2 cm (for consequent de-epithelialization) or 9 x 9 cm pieces (for the cell culture after de-epithelialization).

### HAM de-epithelialization and hAECs isolation

Three different protocols were used for HAM de-epithelialization: 1) incubation with TrypLE Express (Gibco, Grand Island, NY, USA) at 37°C for 10 min; 2) incubation with 0.1% w/v trypsin (Sigma-Aldrich)/0.25% w/v EDTA (Sigma-Aldrich) at 37°C for 30 min; 3) incubation with 125 μg/ml thermolysin (Sigma-Aldrich) at 37°C for 9 min. The incubations were stopped with the Dulbecco´s modified Eagle medium (DMEM; Gibco) containing 10% fetal calf serum (FCS; Gibco), and antibiotics mixture (10 μl/ml of Antibiotic-Antimycotic (100X); Gibco), hereafter referred as the complete DMEM medium. After each de-epithelialization process, HAM pieces were gently scrapped with the cell scraper (Biologix, Shandong, P.R. China) to remove hAECs in sterile petri dish. The medium with cells was collected, centrifuged at 140g for 8 min and resuspended in complete DMEM medium. All experiments were done in duplicates from 8 placentas.

The viability of the hAECs was determined by exclusion of 0.1% w/v trypan blue dye (Gibco) and hAECs were counted with a hemocytometer. De-epithelialized and intact (used as a control) HAMs were frozen in Cryomount (Histolab AB, Askim, Sweden) and stored at -80°C. Tissues were cryosectioned at a thickness of 7 μm, and four slices were mounted per slide.

### Hematoxylin and eosin staining (H&E)

HAMs and HAM cryosections of the control and de-epithelialized HAMs were stained using H&E for the morphological assessment. The samples were examined by light microscopy with the use of Olympus BX51 (Olympus Co., Tokyo, Japan) at a magnification of 100 and 200x.

### DNA analysis

After each de-epithelialization processes, the tissues of size 1 x 1 cm were placed into Eppendorf tube and cut out with scissors. Intact HAM of the same size was used as a control. Tri Reagent (Molecular Research Center, Cincinnati, OH, USA) was added to the tissues, and total DNA was extracted according to the manufacturer´s instructions. The concentration of the DNA was measured with NanoDrop (Thermo Scientific, Waltham, MA, USA).

### Immunostaining

Cryosections of the control and de-epithelialized HAMs from five independent experiments were fixed with iced acetone for 10 min. The samples were incubated with mouse anti-collagen type IV α2 chain (MAB1910; 1:300, Chemicon International, Billerica, MA, USA) or mouse anti- laminin α5 chain antibody (M0638; 1:25, DakoCytomation, Glostrup, Denmark) for one h at room temperature, washed three times with phosphate-buffered saline (PBS) and then incubated with a secondary donkey anti-mouse IgG antibody conjugated with fluorescein (FITC) (715-095-151; 1:200, Jackson ImmunoResearch Laboratories, West Grove, PA, USA). The samples were rinsed with PBS and mounted on slides and DNA counterstained using Vectashield—propidium iodide (Vector Laboratories, Inc. Burlingame, USA). Visualization was performed using Olympus BX51 fluorescence microscope (Olympus Co., Tokyo, Japan) at a magnification of 200x. Images were recorded using a Vosskühler VDS CCD-1300 camera, (VDS Vosskühler GmbH, Germany), and NIS Elements software (Laboratory Imaging, Czech Republic) was used for image analysis.

### Scanning electron microscopy (SEM)

Samples of intact and denuded HAM scaffolds (from two placentas) mounted in a CellCrown™ inserts (Scaffdex Oy, Tampere, Finland) were fixed in PBS buffered 3% glutaraldehyde, washed in PBS, postfixed with 1% OsO4, dehydrated in a graded ethanol series (25, 50, 75, 90, 96, and 100%) and critical point dried in a K850 Critical Point Dryer (Quorum Technologies Ltd, Ringmer, UK). The dried samples were sputter-coated with 3 nm of platinum in a Q150T Turbo-Pumped Sputter Coater (Quorum Technologies Ltd, Ringmer, UK). The final samples were examined in a FEI Nova NanoSEM scanning electron microscope (FEI, Brno, Czech Republic) at 5 kV using ETD, CBS and TLD detectors. Stereo-pair images were taken at tilts of -6°, 0° and +6° of compucentric goniometer stage. Final R-GB anaglyphs were constructed in a “Stereo module” of AnalySis3.2 software suite (EMSIS GmbH, Germany).

### Cell culture

The hAECs harvested from three placentas after trypsin/EDTA de-epithelialization from 9 x 9 cm HAM pieces were cultured in complete DMEM medium in 25-cm^2^ tissue culture flasks (Techno Plastic Products, Trasadingen, Switzerland). Medium was changed every 3–4 days. When the cell culture confluence reached about 80–90%, the cells were passaged with 1 ml of TrypLE Express for 5 min in 37°C. The hAECs were collected, centrifuged at 140g for 8 min and counted with hemocytometer. After every passage, the cells (10 x 10^3^ cells) were used for the WST-1 assay, approximately 100 x 10^3^ cells were transferred to the Eppendorf tubes with Tri Reagent (Molecular Research Center, Cincinnati, OH) and one third of the cells were put back to the culture flask and cultured to the next confluence and passage. The cell images were taken before each passage, and similarly the metabolic activity and gene expression of the cells was determined.

### Determination of metabolic cell activity

The metabolic activity of living cells was determined by the WST-1 assay as we described before [45]. In brief, the hAECs (10 x 10^3^ cells) were cultured in complete DMEM medium with or without epidermal growth factor (EGF) (Gibco) in 96-well tissue culture plate (VWR, Radnor, PA, USA) for 24 h at 37°C in an atmosphere of 5% CO2. WST-1 reagent (Roche, Mannheim, Germany) (10 μl/100 μl of the medium) was added to each well, and the plates were incubated for another 4 h to form formazan [46]. Formazan-containing medium (100 μl) was transferred from each well into the new 96-well tissue culture plate and the absorbance was measured using a Tecan Infinite M200 (Tecan, Männedorf, Switzerland) at a wave-length of 450 nm.

### Isolation of RNA and reverse transcription PCR (RT-PCR)

The cells were transferred into Eppendorf tubes containing 500 μl of TRI Reagent and total RNA was extracted according to the manufacturer´s protocol as was described previously [47]. RNA quality was analyzed by λ260/λ280 spectrophotometer analysis (Nanodrop). One μg of RNA was treated with deoxyribonuclease I (Promega, Madison, WI) and used for subsequent reverse transcription. The first-strand cDNA was synthesized using random hexamers (Promega) in a total reaction volume of 25 μl using M-MLV Reverse Transcriptase (Promega).

The first strand cDNA product (2μl) was amplified in a total volume of 20 μl PCR assay, containing 10 μl PPP Master Mix (Top Bio, Vestec, Czech republic), 1 μl of each primer and was filled up to a total volume with PCR water (Top Bio). The primers for *β-ACTIN*, *SOX2*, *OCT-4*, *OCT-4A* and *NANOG* were selected from previous works and specificity was examined with Primer-BLAST software (NCBI) [20, 39, 48]. Two pairs of primers for *OCT-4* were used, because there are two possible spliced variants (*OCT-4A* and *OCT-4B*). Product sizes, annealing temperatures and primer sequences are listed in Table 1. The PCR cycles included denaturation at 94°C for 2 min followed by 35 to 40 cycles as follows: denaturation at 94°C for 30 s, annealing 57°C to 64°C for 30 s, elongation at 72°C for 1 min and 72°C extension for 10 min at the end of the program. RT-PCR products were visualized with Gel Red (Biotium, CA, USA) on a 1% agarose gel. Amplification of the housekeeping gene *β-ACTIN* transcripts was performed simultaneously in order to confirm RNA integrity. Induced pluripotent stem cells (iPS) were used as positive control and corneal fibroblasts as negative control for expression of stem cell markers. Both cell types were prepared as was described previously [49, 50]. Non template control (NTC) reactions were used without cDNA.

**Table 1:** Primer sequences used for real-time PCR.

Primer	Sequence (5´-3´)	Product size (bp)	Annealing temperature (°C)	Cycles	References
β-ACTIN	F: cgcaccactggcattgtcat R: ttctccttgatgtcacgcac	208	57	35	[20]
SOX2	F: gccgagtggaaacttttgtc R: gttcatgtgcgcgtaactgt	264	57	40	[20]
NANOG	F: ctgtgatttgtgggcctgaa R: tgtttgcctttgggactggt	153	57	35	[39]
OCT-4	F: gaggagtcccaggacatgaa R: gtggtctggctgaacacctt	151	57	40	[20]
OCT-4A	F: cttctcgccccctccaggt R: aaatagaacccccagggtgagc	496	64	35	[48]

### Statistical analysis

The statistical significance of differences between individual groups was calculated using the Student’s t-test.

## Results

### De-epithelialization of HAM and BM integrity

**Fig 1:**
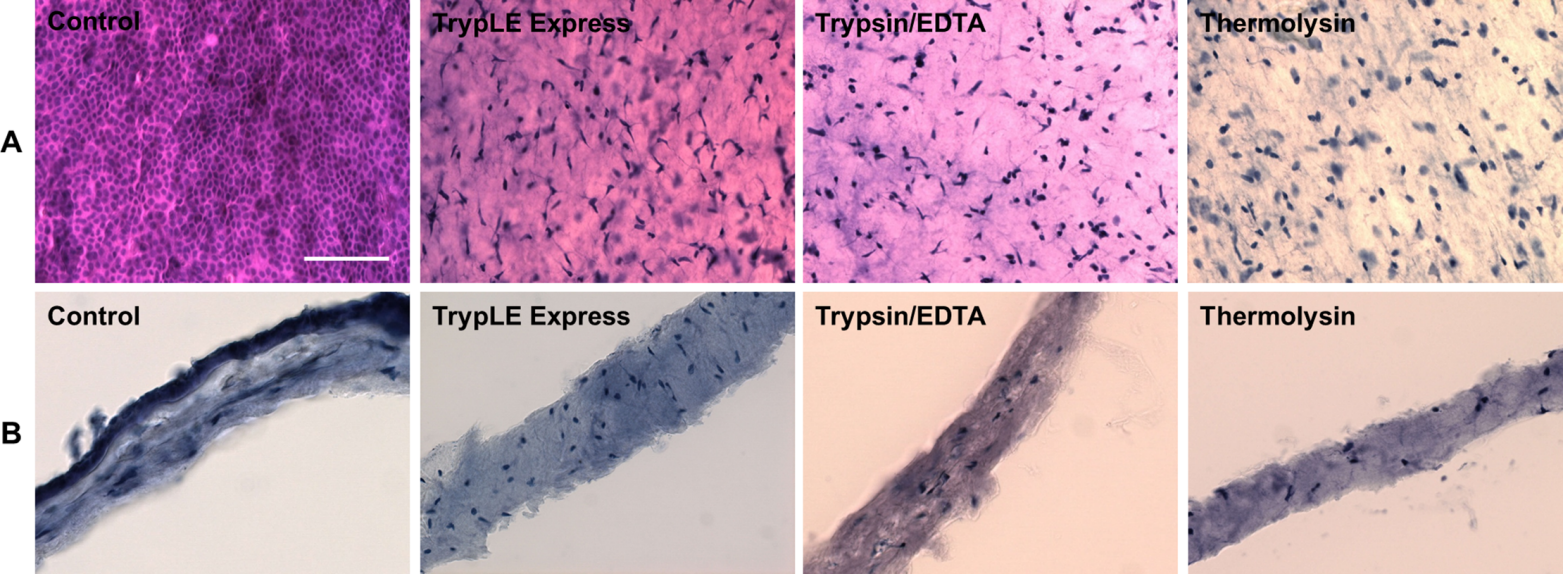
Comparison of the intact and denuded HAMs and HAM cryosections. Comparison of the intact (Control) and denuded HAMs (A) and HAM cryosections (B) after TrypLE Express, trypsin/EDTA and thermolysin treatment stained with H/E for light microscopy. Scale bar represents 100 μm.

The integrity of HAM, the quality of de-epithelialization, and potential presence of hAECs were verified by H&E staining and SEM analysis. The surface of intact HAM consists of cuboidal epithelial cells, mesenchymal cells were observed scattered in the stroma (Fig 1).

All three enzymatic methods (TrypLE Express, trypsin/EDTA, and thermolysin) were comparable in term of efficiency of HAM de-epithelialization. Only few epithelial cells occasionally rested on denuded HAM with no difference of the used treatment. The hAMSCs from non-treated HAM exhibited spindle-shaped morphology, similarly as hAMSCs after TrypLE Express and trypsin/EDTA treatments. The thermolysin application led to loss of mesenchymal spindle-shaped cell morphology, showing rather round cell shape (Fig 1).

**Fig 2:**
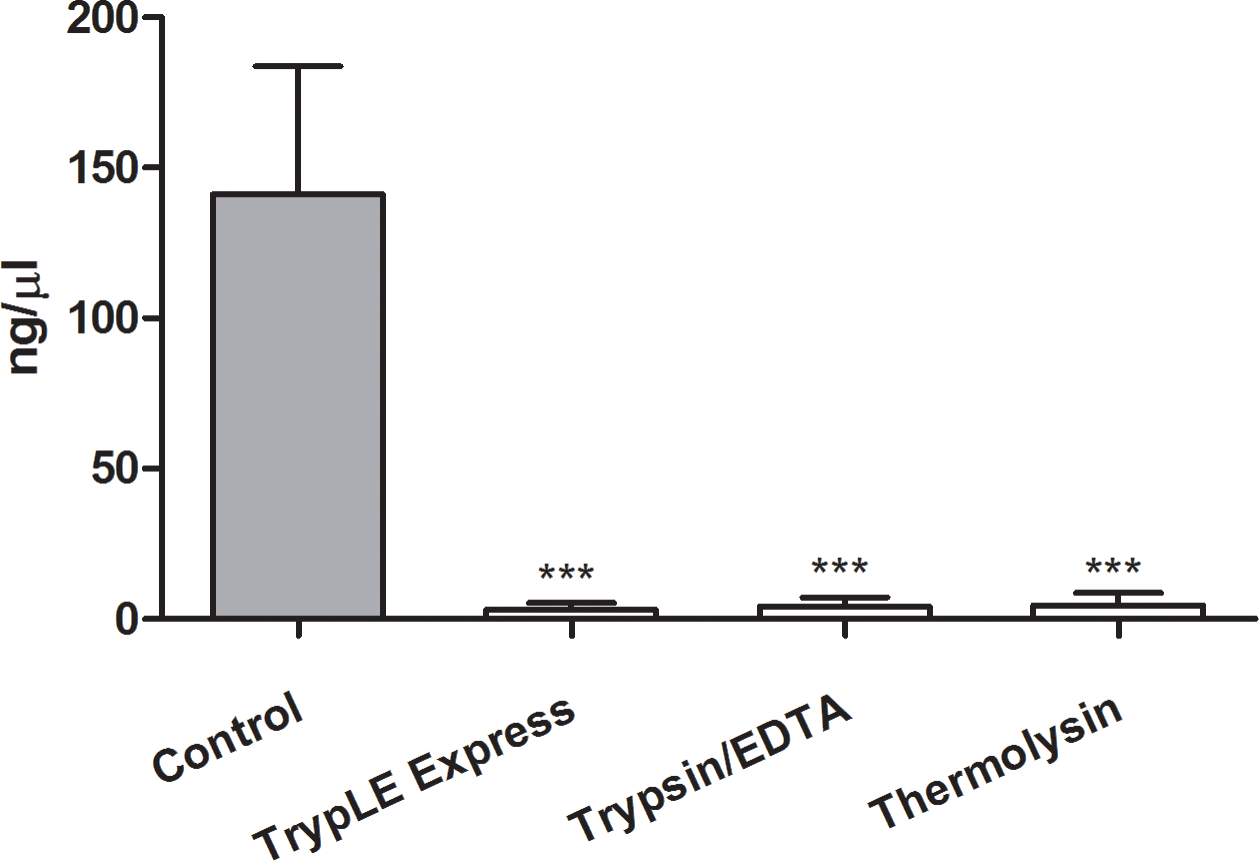
Comparison of the DNA concentrations. Comparison of the DNA concentration in the tissues form the intact (Control) and denuded HAMs with TrypLE Express (TrypLE), trypsin/EDTA and thermolysin treatment directly after de-epithelialization. Each bar represents mean ± SD from 3 determinations (***P < 0.001).

The DNA concentration in denuded HAM was significantly lower after the treatment with all de-epithelialization agents compared to control untreated samples (Fig 2). The small residual amount of DNA in treated specimens represents DNA of hAMSCs.

**Fig 3:**
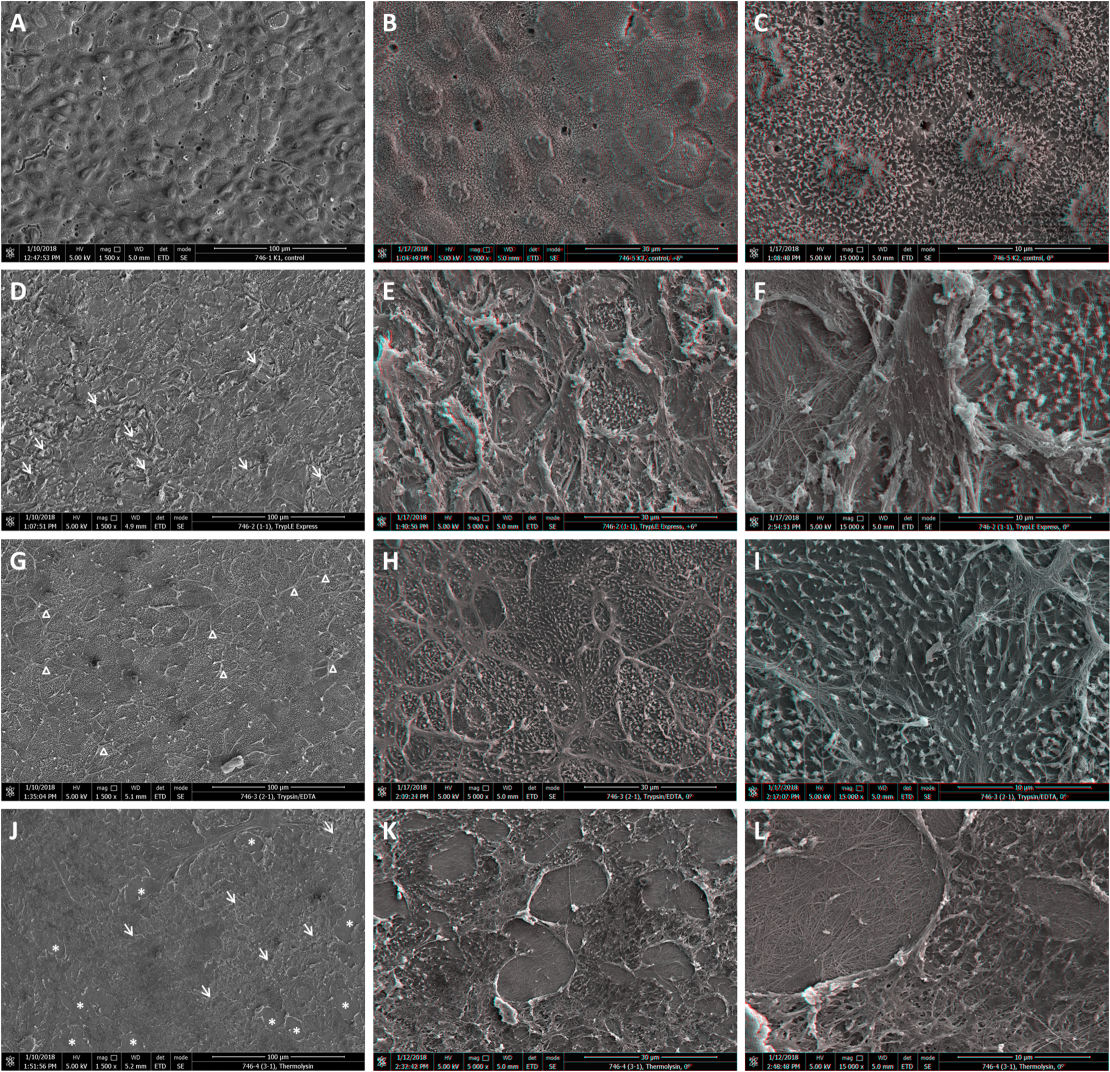
Topography of intact and denuded HAM. Scanning electron micrographs (A, D, G, J) and stereo anaglyphs (B, C, E, F, H, I, K, L) of the intact (A, B, C) and denuded HAM by TrypLE Express (D, E, F), trypsin/EDTA (G, H, I) and thermolysin (J, K, L). Areas of damaged BM are marked by arrows, ruptured gaps by *, the residues of ECM by Δ. Red-green or red-cyan glasses required for proper view of stereo anaglyphs.

The mosaic layer of hAECs covered with dense microvilli was determined at the surface of intact HAM by SEM analysis (Fig 3A, 3B and 3C). BM is well preserved after trypsin/EDTA treatment, some residues of extracellular matrix (ECM) from epithelial cell layer are clearly detectable (Fig 3G, 3H and 3I). Partial damage of BM was observed after applying TrypLE Express treatment, but BM stayed still mostly intact (Fig 3D, 3E and 3F). When thermolysin was used for decellularization, the BM was damaged and numerous lesions were observed revealing the collagen network of compact layer under BM (Fig 3J, 3K and 3L), suggesting aggressive proteolysis.

**Fig 4:**
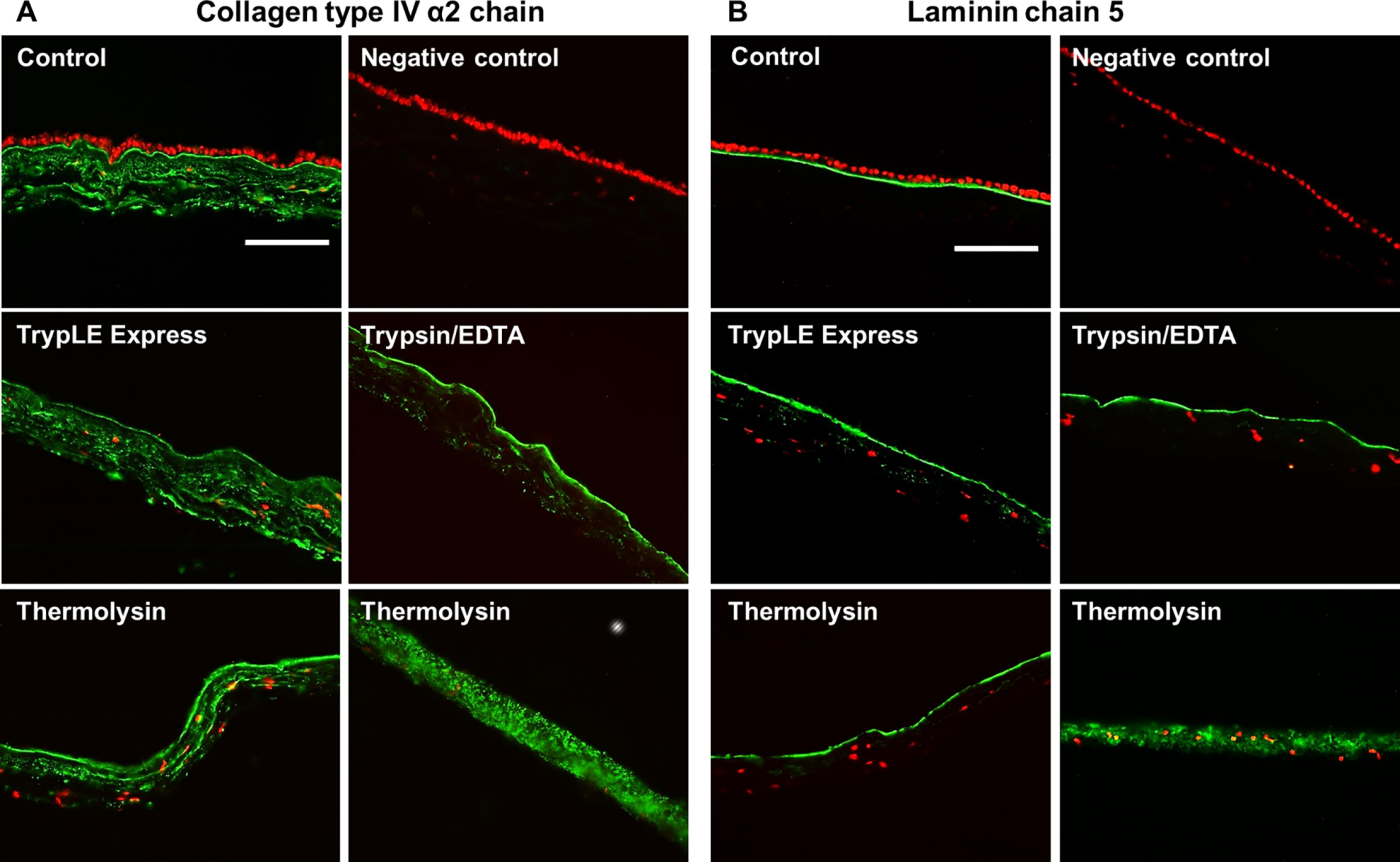
Immunostaining of BM. Distribution of BM collagen type IV α2 chain (green; A) or laminin α5 (green; B) in intact (Control) and denuded HAM: TrypLE Express, trypsin/EDTA, thermolysin treatment. Intact HAM (primary antibody omitted), was used as negative control. Cell nuclei were stained with the propidium iodide (red). Scale bar represents 100 μm.

Collagen type IV and laminin α5 chain showed clear positivity in BM of all control specimens and specimens after TrypLE Express and trypsin/EDTA treatment (Fig 4). After thermolysin application, two staining patterns were observed: in HAM specimens from three placentas, the staining for both proteins was properly localized just in BM without any visible integrity deterioration, on the other hand, the positive signal of collagen type IV and laminin α5 was spread throughout the whole amniotic stroma in specimens from other two placentas. In these samples the positive line representing BM was not apparent (Fig 4A and 4B). Intact HAM was used as a negative control without using primary antibody.

### Viability, morphology, growth and expression pattern of hAECs

**Fig 5:**
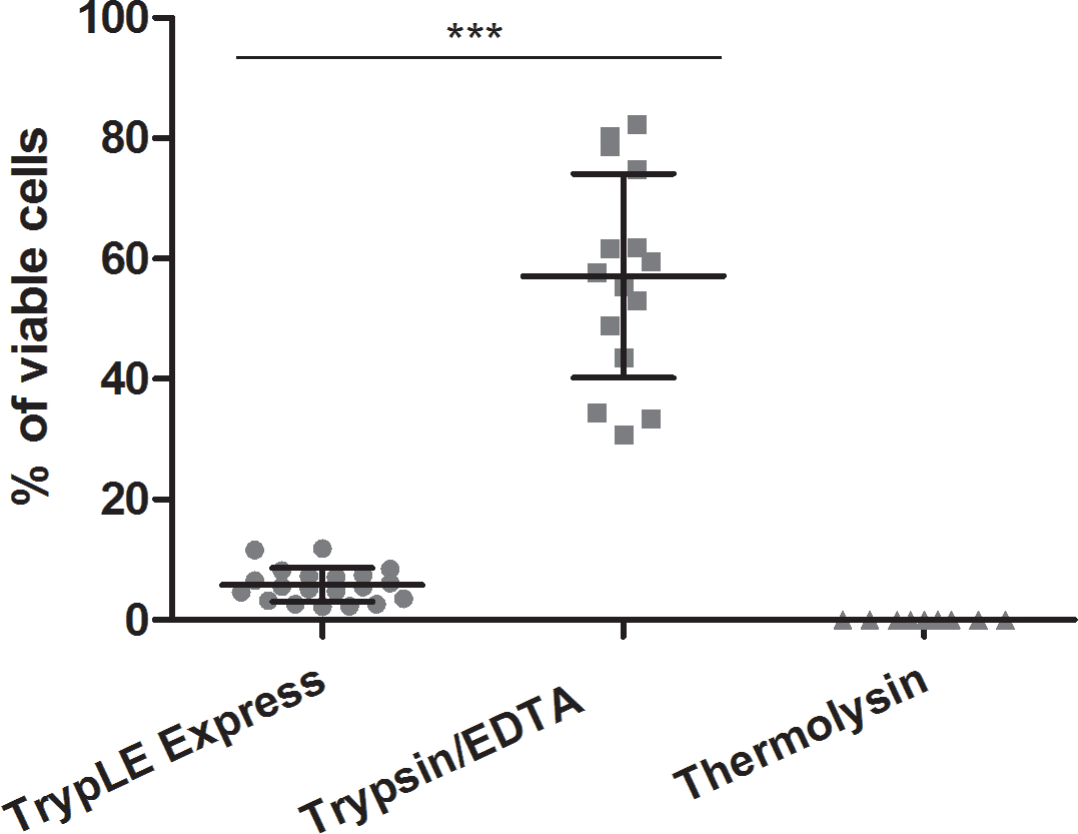
The viability of hAECs. Comparison of the hAECs viability after TrypLE Express, trypsin/EDTA and thermolysin treatment. Cells were stained with trypan blue and counted via hemocytometer. Each bar represents mean ± SD from 15 determinations (***P < 0.001).

The viability of obtained hAECs immediately after de-epithelialization reached approximately 6% after TrypLE Express, and about 60% after trypsin/EDTA treatment (Fig 5). Only dead cells and cellular fragments were observed after de-epithelialization using thermolysin.

**Fig 6:**
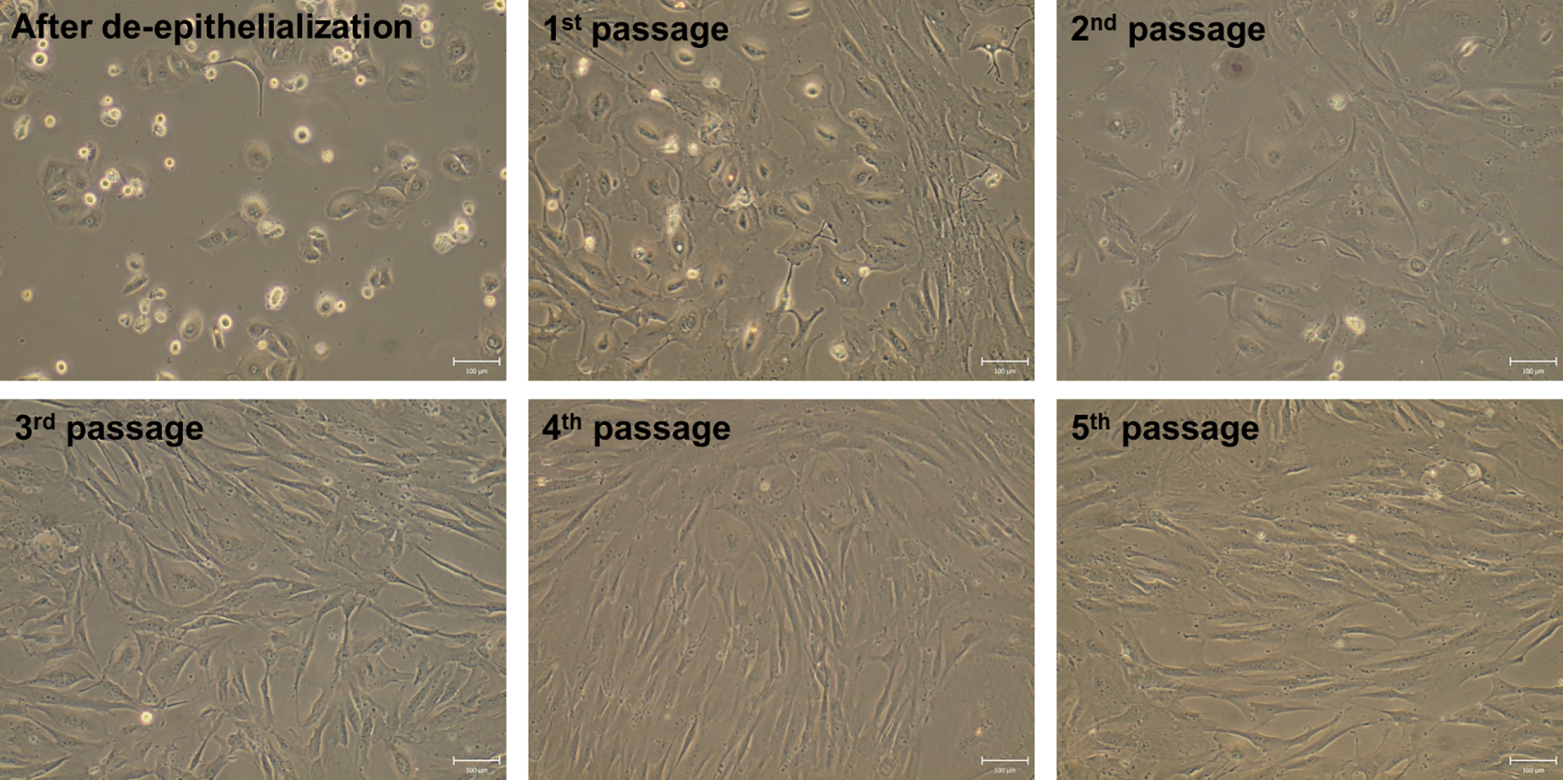
The morphology of hAECs. The comparison of morphology of cultured hAECs after trypsin/EDTA treatment in complete DMEM medium. The cells for the light microscopy were photographed before each passage (after de-epithelialization, before 1^st^, 2^nd^, 3^rd^, 4^th^ and 5^th^ passage). Results of one out of 3 identical experiment is shown. Scale bars represent 100 μm.

**Fig 7:**
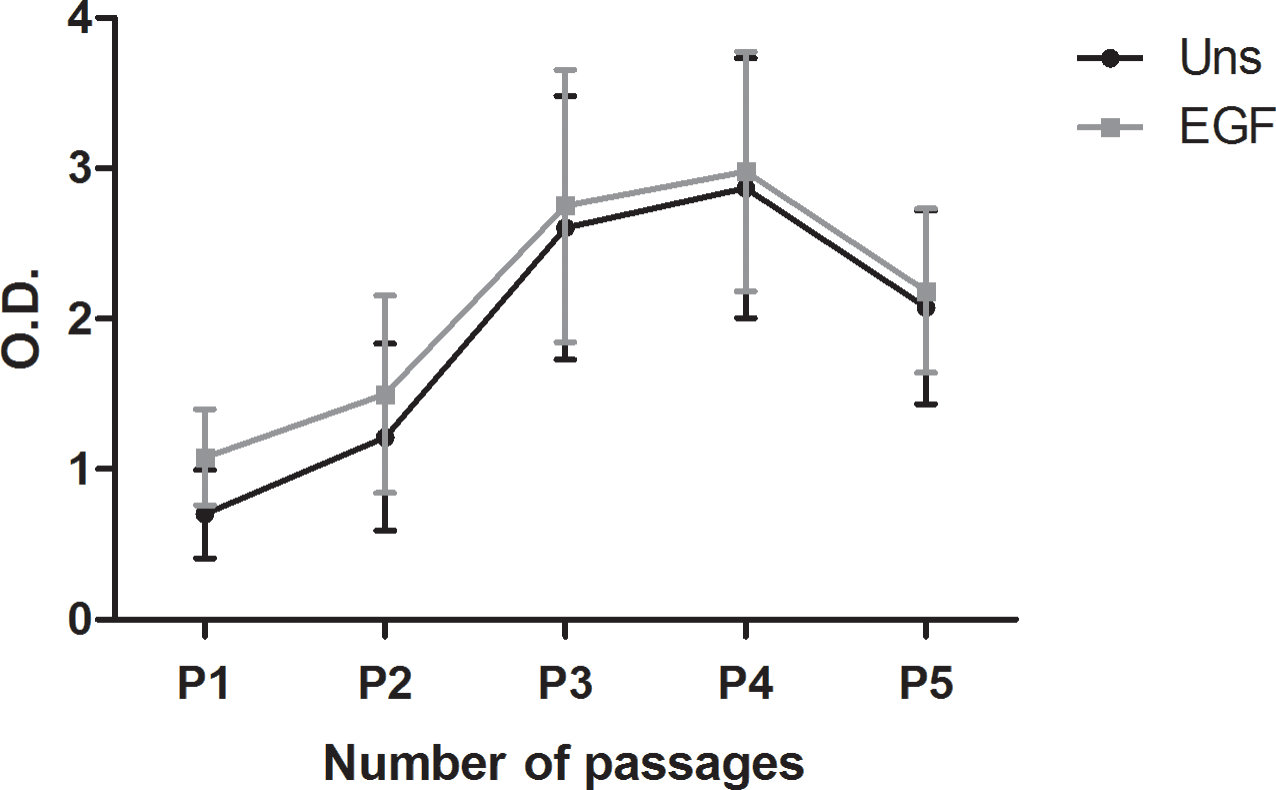
The metabolic activity of hAECs. Comparison of metabolic activity of the epithelial cells unstimulated (Uns) and stimulated with EGF (EGF) after each passage. WST-1 reagent was added to the cell cultures for 4 h to form formazan. The absorbance was measured at a wave-length of 450 nm. Each bar represents mean ± SD from 3 determinations.

The hAECs harvested after trypsin/EDTA treatment were successfully cultured from all three HAMs. The morphology of hAECs changed from cuboidal shape at the beginning of the culture to more mesenchymal shape cells in the 4^th^ and 5^th^ passage (Fig 6). The higher proliferation activity was observed in later passages. When hAECs were co-cultured with EGF for 24 hours, the metabolic activity was slightly, but not significantly increased (Fig 7).

**Fig 8:**
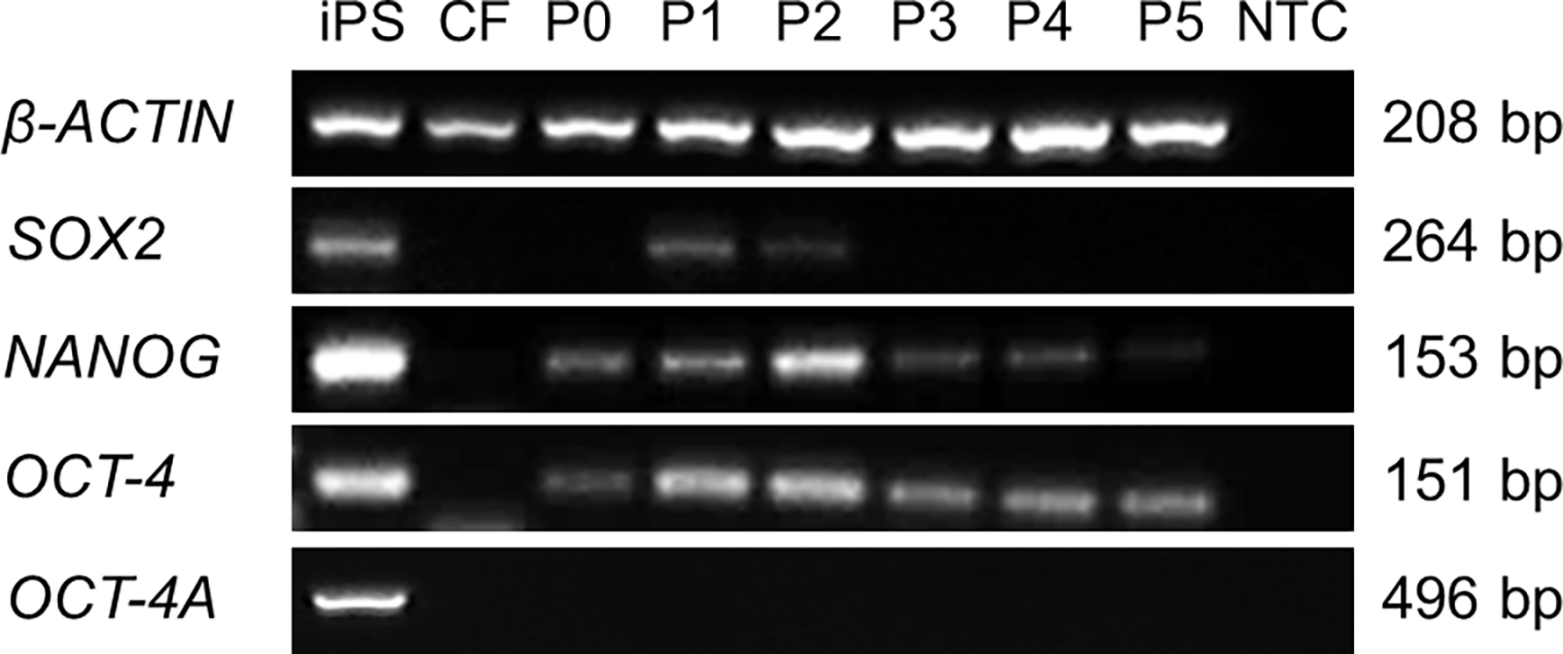
The RT-PCR analysis of hAECs. The RT-PCR analysis of hAECs after de-epithelialization and each passage (P0-P5). The iPS cells were used as a positive (iPS) and corneal fibroblasts as negative control (CF). Sample without cDNA (NTC) was used as non-template control. One representative experiment of 3 (with identical results) is shown.

The expression of three stem cell markers in cultured hAECs was detected. *SOX2* was present up to 2^nd^ passage, *NANOG* up to 4^th^ passage, and *OCT-4* was present in all passages (Fig 8). No band was observed when primers for transcription variant specific for stem cells (OCT-4A) were used.

## Discussion

The three tested de-epithelialization approaches were efficient to remove epithelial cells from HAM surface. However, only the treatment with trypsin/EDTA was effective for simultaneous harvesting of viable hAECs. We have shown, that gentle mechanical scrapping necessary to remove up to 100% of hAECs after each treatment does not affect the integrity of BM. The staining of HAM and DNA concentration measurement demonstrated the efficiency of all three de-epithelialization processes, with no significant difference between the methods.

On the other hand, the detections of collagen type IV and laminin α5 as ubiquitous components of BM [51, 52] revealed some differences between used protocols. The regular staining of BM after TrypLE Express and trypsin/EDTA treatment indicates its integrity and is in agreement with previously published data [13]. We have shown that relatively low trypsin concentration (0.1% w/v) in trypsin/EDTA mixture does not affect BM integrity and cell vitality. BM degradation has been documented after treatment with higher (0.25% w/v) trypsin concentration [13]. The results from SEM analysis thoroughly confirm our original conclusions based on histology and immunohistochemistry data. Smooth surface and the presence of BM after trypsin/EDTA treatment were also already detected [13]. In our experiments only partial damage of BM has been noticed when TrypLE Express was used.

Different situation was observed after de-epithelialization using thermolysin, where almost 50% of specimens showed, beside integral BM staining, signal of collagen type IV and laminin α5 dispersed in HAM stroma. Also loss of hAMSCs spindle shape morphology is consistent with damages induced by thermolysin. SEM analysis showed that BM was damaged and ruptured. The collagen fibres of the underlying compact layer were seen at locations where BM was missing. The similar image of collagen fibres was observed after de-epithelialization by dispase when entire BM was absent [13]. Thermolysin is a heat-stable metalloproteinase which acts specifically at hemidesmosome complex at the level of BM [53], most likely targeting collagen IV but not laminin [53, 54]. Hopkinson et al. also noted certain damage of BM when thermolysin in combination with mechanical scrapping was used [33]. The improvement of BM integrity was achieved, when mechanical removal was replaced by simple washing [33]. Unfortunately, we were unable to denude the HAM completely with thermolysin only. The fragility and difficult handling of HAM after thermolysin treatment has been also reported in another study [34]. We consider that the damage of the BM is caused by the natural activity of this enzyme due to cleavage of collagen IV. Moreover the lesions are often of round or oval shape (see Fig 3K and 3L), but not cracks, as it would correspond to scrapping damage.

De-epithelialization using thermolysin resulted in complete loss of hAECs viability. On the other hand thermolysin was successfully used for the isolation of epidermal or intestinal epithelial cells [53, 55], which are probably less sensitive to enzymatic treatment than the hAECs.

The highest viability of hAECs (about 60%) after trypsin/EDTA indicates that this method is gentle and safe. We have also tried to culture hAECs harvested after TrypLE Express method (6% viability), but these cells (probably due to low initial amount of cells) were growing very slowly and reached full confluence only after extended time periods. The viability of hAECs after TrypLE Express treatment did not changed even if we used a prolonged time period (30 min). The hAECs obtained by trypsin/EDTA treatment were successfully cultured up to 5^th^ passage and their proliferation activity increased after each passage up to the 4^th^ one. It was reported that addition of EGF as mitogenesis promoter [56] significantly increases proliferative capacity of hAECs [41]. The addition of EGF for to 24-h culture period did not change proliferation activity significantly. The longer cultivation periods in our study was omitted as it has been found that 7-day cultivation of hAECs with EGF led to significantly increased proliferation, but lower expression of pluripotent genes *OCT-4*, *SOX2* and *NANOG* [57]. Our hAECs, isolated with trypsin/EDTA method, changed their morphology during culture and passaging from more cuboidal morphology at the beginning of culture to more mesenchymal shape from the 3^rd^ passage. Similar observation was also described repeatedly [36]. Morphology and proliferation changes could be caused by epithelial to mesenchymal transition by autocrine production of transforming growth factor-β during the culture of hAECs [58].

It has been shown that hAECs express molecular markers of pluripotent stem cells: *NANOG*, *SOX2* and *OCT-4* [20, 39]. We detected the expression of *NANOG* in cells after de-epithelialization and throughout cultivation; *SOX2* was present in two first passages only. The detection of *OCT-4* was more complex due to its nature. *OCT-4* plays a crucial role in regulating the self-renewal and maintaining pluripotency [59, 60] and encodes two main variants known as *OCT-4A* and *OCT-4B* [61]. While the expression of *OCT-4A* is restricted to embryonic stem cells and embryonal carcinoma cells, *OCT-4B* can be detected in various nonpluripotent cell types [48, 62, 63]. In recent studies some authors still used the primers fitted on both variants for PCR analysis [39, 57, 64]. Using primers suitable for both variants, we detected expression of *OCT-4* in each passage, but *OCT-4A* spliced variant (primers selected based on the work of Atlasi et al. [48]), was not detected in any passage of the cells. On the contrary, Izumi et al. confirmed *OCT-4A* expression in naive (but not cultured) hAECs by using a commercially available primer and probe set that matches *OCT-4A* specific exons by quantitative RT-PCR [65]. In summary, our data on detection of expression of pluripotent stem cell markers suggest that stemness of cultured hAECs decreases with each passage.

Out of three tested de-epithelialization protocols (TrypLE Express, trypsin/EDTA, thermolysin) trypsin/EDTA application showed to be the most efficient when both viable hAECs and intact BM are requested. We would like to stress here, that the term “intact” is used for visibly least damaged BM (judged by the SEM analysis) where no observable lesions were detected contrary to BM obtained by other two methods (see Fig 3). This does not necessarily mean, that some eventual minor structural modification do not occur during trypsin/EDTA treatment (e.g. collagen fiber structure modification), however, these have not an impact on the integrity of BM. The major goal of this study was to establish the conditions under which both undamaged BM and viable hAECs can be obtained and our results demonstrate, that the trypsin/EDTA treatment is the most efficient approach. It leads to successful de-epithelialization of HAM with undamaged BM with well-preserved integrity and at the same time to harvesting of viable epithelial cells which can be cultured up to 5^th^ passage with gradually increasing proliferation capacity. The stemness properties of these cells, however, decrease with higher passages. The cell viability, on the other hand also correlates well with level of BM damage. The method which yields no viable cell (thermolysin) also provides BM with most profound lesions, while intact BM (Trypsin/EDTA, Fig 3G, 3H and 3I) correlates with the best viability of harvested cells (Fig 4). Therefore, we suggest that the trypsin/EDTA method is the method of choice when both intact HAM and viable hAECs are needed for subsequent use.
